# Attenuation of LPS-induced inflammatory responses in J774A.1 macrophages by phenylpropanoids and ursane triterpenes from *Lavandula coronopifolia* Poir.

**DOI:** 10.1038/s41598-026-51849-5

**Published:** 2026-05-24

**Authors:** Marwa Elsbaey, Eman Elattar, Álvaro Mourenza, Pablo Castañera, Luis M. Mateos, Michal Letek, Mai H. ElNaggar

**Affiliations:** 1 Department of Pharmacognosy, Faculty of Pharmacy, Mansoura University, 25 El Gomhouria St, Mansoura, Dakahlia Governorate 35516 Egypt; 2 Departamento de Biología Molecular, Área de Microbiología, Universidad de León, León, 24071 Spain; 3 Department of Pharmacognosy, Faculty of Pharmacy, Kafrelsheikh University, Kafrelsheikh, 33511 Egypt; 4 Instituto de Desarrollo Ganadero y Sanidad Animal (INDEGSAL), Instituto de Investigación Biosanitaria de León (IBIOLEÓN), Campus Universitario Vegazana, León, 24071 España; 5 Marine Biodiscovery Centre, Department of Chemistry, School of Natural and Computing Sciences, University of Aberdeen, Old Aberdeen, AB24 3UE UK

**Keywords:** *Lavandula coronopifolia*, Triterpenes, Scratch wound assay, IL-6, iNOS, Biochemistry, Cancer, Cell biology, Drug discovery

## Abstract

Phytochemical investigation of the non-volatile constituents of *Lavandula coronopifolia* Poir has led to the isolation of seven compounds (**1**–**7**). 2α, 3*β*, 23-Trihydroxyurs-12,18-dien-28-oic acid 28-*O-β*-d-glucopyranoside (**5**) exhibited selective cytotoxic activity against A549 lung carcinoma, with EC_50_ value of 11.5 µM, and showed no toxicity towards the normal HEK293T cells. The anti-inflammatory potential of **1**–**7** was assessed in lipopolysaccharide (LPS)-stimulated J774A.1 cells. Methyl rosmarinate (**2**), 1*β*, 2*α*, 3*β*, 19*α*, 23-pentahydroxy-urs-12-en-28-oic acid-28-*O*-*β*-d-glucopyranoside (**3**) and 2*α*, 3*β*, 23-trihydroxyurs-12, 19-dien-28-oic acid 28-*O*-*β*-d-glucopyranoside (**6**) effectively reduced cell migration as revealed by the scratch wound assay. They also altered cell morphology in a manner similar to dexamethasone. Furthermore, qPCR revealed that **2**, **3** and **6** significantly downregulated the expression levels of nitric oxide synthase (iNOS) and interleukin-6 (IL-6) as compared to LPS-stimulated J774A.1 cells. The results highlight the potential of **2**, **3** and **6** for anti-inflammatory therapies and **5** as a candidate for lung cancer.

**Supplementary Information:**

The online version contains supplementary material available at 10.1038/s41598-026-51849-5.

## Introduction

Inflammation is a fundamental biological response that protects the body against tissue injury and infection^1,2^. However, when this response becomes chronically sustained, it shifts from a protective mechanism to a central pathogenic driver underlying numerous conditions, including cardiovascular, metabolic, renal, autoimmune, and neurodegenerative diseases, as well as many cancers and fibrotic organ failure^1,2^. Natural products continue to represent a rich and indispensable source for the discovery and development of novel therapeutics targeting diseases driven by chronic inflammation^3^.

The genus *Lavandula*, commonly known as lavender, includes approximately 47 species of aromatic flowering plants belonging to the family Lamiaceae. In addition to their wide pharmaceutical applications, lavenders are widely used in the field of cosmetics, foods, and aromatherapy^4^. Lavanders have been used in folk medicine for the treatment of pain, headache, migraine, diabetes, epilepsy, rheumatism, and as carminatives^5^.

*Lavandula coronopifolia* Poir. is a shrub-like perennial that grows in sandy and gravel soils^6^. It is described as Saharo-Arabian, extending its distribution to the Eastern Sudanian territories^6^. The essential oil of *L. coronopifolia* is reported to possess antibacterial^7^, cytotoxic^8^, anti-inflammatory^9^ and neuroprotective^10^ activities. Few phytochemical studies have addressed the non-volatile constituents of *L. coronopifolia* reporting the presence of flavonoids^6^, pentacyclic triterpenes^11,12^, caffeic acid and rosmarinic acid^5^. Pharmacological studies have demonstrated the antioxidant, antimicrobial, hepatoprotective and α-glucosidase inhibitory activities of *L. coronopifolia*^4,5,11^.

The essential oils of lavenders including *L. coronopifolia*^9,10^ are well documented for their anti-inflammatory^13^ and cytotoxic potential^8,14^. While the majority of phytochemical studies have focused on the volatile constituents, the non-volatile metabolites have not been thoroughly explored. Apart from one study investigating the cytotoxic potential of the ethyl acetate fraction of *L. coronopifolia*^15^, the cytotoxic and anti-inflammatory potential of its non-volatile compounds remains underexplored. Moreover, focusing exclusively on volatile compounds may overlook potentially potent bioactive molecules that contribute to or even synergize the observed pharmacological effects of the volatile components. Investigating the non-volatile fraction thus provides a more comprehensive understanding of the plant’s chemical profile and therapeutic potential.

Accordingly, this study aimed to purify, characterize and asses the major non-volatile constituents of *L. coronopifolia* for their anticancer and anti-inflammatory potential. This approach not only complements existing knowledge on the plant’s essential oils but also expands the scope of its pharmacological relevance by identifying additional classes of bioactive compounds and maximizing the plant utilization.

## Results and discussion

### Identification of the isolated compounds

Phytochemical investigation of *L. coronopifolia* has led to the isolation of two phenylpropanoid derivatives and five ursane triterpenes (Fig. 1). Based on comparison of their NMR and LC–MS data (Figs. S1–S28) to the literature, they were identified as follows: caffeic acid (**1**)^16^; methyl rosmarinate (**2**)^17^; 1*β*, 2*α*, 3*β*, 19*α*, 23-pentahydroxy-urs-12-en-28-oic acid-28-*O*-*β*-d-glucopyranoside (**3**)^12^; nigaichigoside F1 (**4**)^18^; 2α, 3*β*, 23-trihydroxyurs-12,18-dien-28-oic acid 28-*O-β*-d-glucopyranoside (**5**)^19,20^; 2*α* ,3*β* ,23-trihydroxyurs-12,19-dien-28-oic acid 28-*O*-*β*-d-glucopyranoside (**6**)^21^; and quadranoside VIII (**7**)^21^. It is worth to note that **1**–**4**^5,11,12^ were previously isolated from *L. coronopifolia*; while **5**–**7** are reported for the first time.

**Fig. 1:**
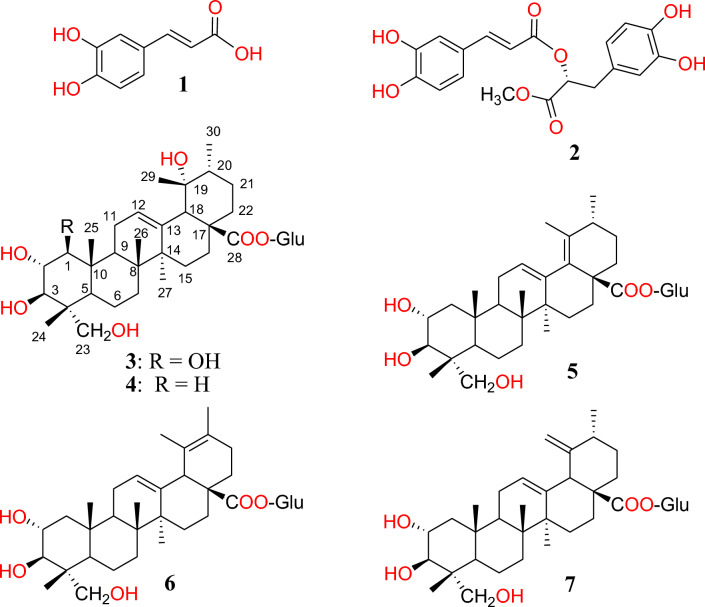
Structures of compounds isolated from *Lavandula coronopifolia* Poir.

### Cytotoxic activity

The volatile oil of *L. coronopifolia* has been reported to exhibit cytotoxic activity against several human cancer cell lines^8,9^. Additionally, different organic solvent extracts of *L. coronopifolia* aerial parts exhibited hepatoprotective activity by mitigating ethanol-induced oxidative stress and cytotoxicity in HepG2 cells^22^. To further assess the biological potential of *L. coronopifolia*, both the cytotoxic and anticancer activities of the isolated compounds were evaluated. The anticancer activity was examined against the human lung carcinoma cell line A549, while the human embryonic kidney cell line HEK293T was used as a non-cancerous control to assess cytotoxicity, using phosphate-buffered saline (PBS) as a negative control.

**Fig. 2:**
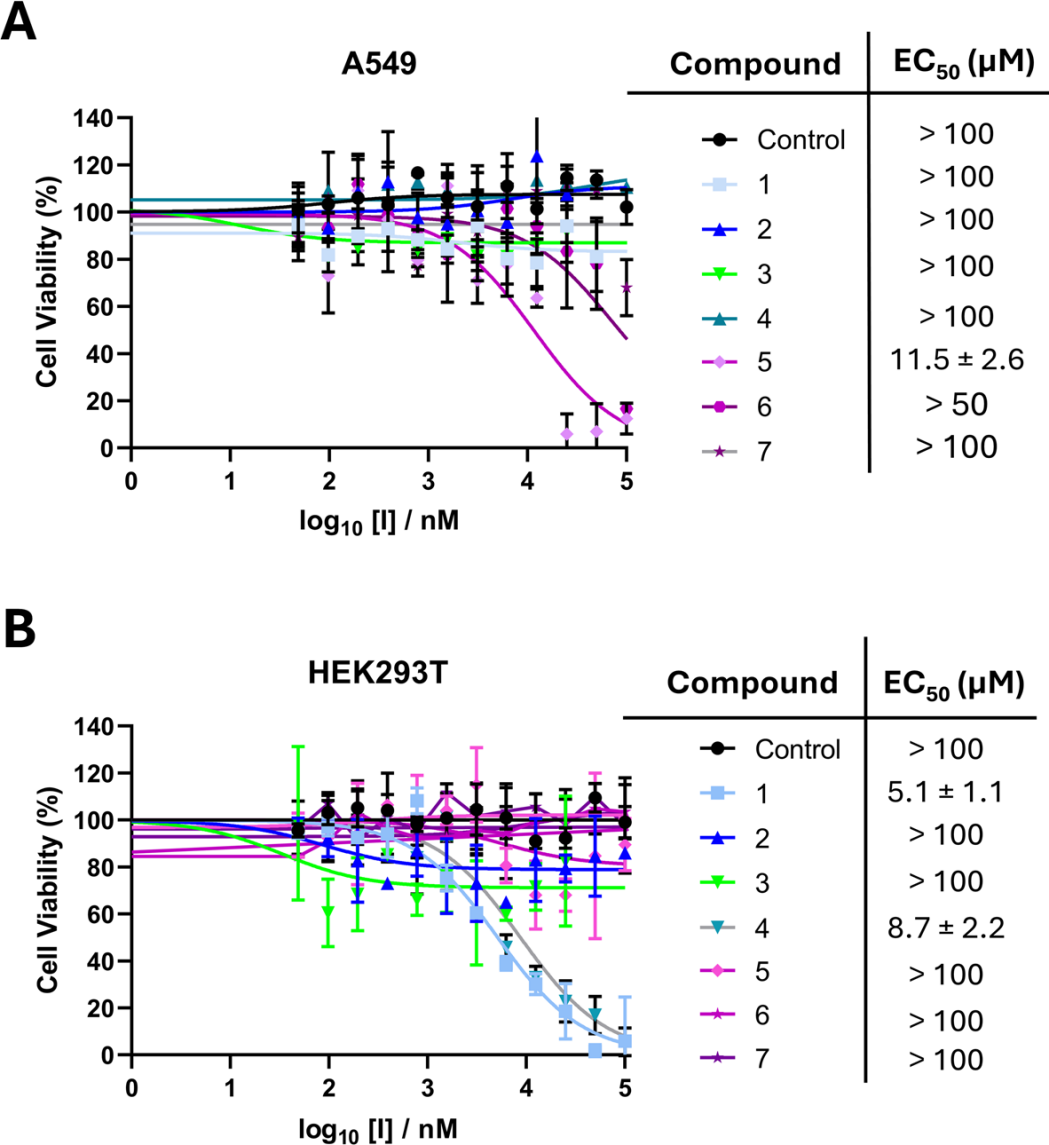
Cell viability (MTT) assay of (**A**) lung carcinoma (A549) cells, and (**B**) human embryonic kidney cells (HEK293T) exposed to different concentrations (0–100 µM) of compounds (**1**–**7**) after 48 h, using PBS as a negative control. Data are presented as mean ± SD, experiments were conducted in triplicate.

Among the compounds tested, only compound **5** exhibited anticancer activity against A549, with an EC₅₀ value of 11.5 µM (Fig. 2A), while showing no cytotoxicity towards HEK293T cells at > 100 µM, indicating a good therapeutic index. Interestingly, its isomers, compounds **6** and** 7**, showed no cytotoxicity, suggesting that the presence of a double bond between carbons 18 and 19 in the ursane nucleus may be crucial for anticancer activity.

In contrast, compounds **1** and **4** demonstrated marked cytotoxic activity against HEK293T cells with EC₅₀ values of 5.1 and 8.7 µM, respectively (Fig. 2B) but showed no inhibitory effect on A549 cells. Their selective cytotoxicity towards the non-cancerous cells suggests that compounds **1** and **4** are poor therapeutic candidates. Comparing the cytotoxicity of the isomers **3** and **4**, it can be concluded that the presence of OH group at C-1 of the ursane nucleus may modulate the compound toxicity against normal HEK293T cells.

### Anti-migration activity using scratch wound assay (2D migration assay)

*Lavandula* members have been widely used in traditional medicine across the Mediterranean, Middle East, and parts of Asia for treating inflammation and related conditions such as headaches, skin irritations, and pain. Ethnomedicinal records document the use of various *Lavandula* species for inflammatory aliments, both topically and internally^23–25^. Modern studies suggest that their significant anti-inflammatory activity is attributed to essential oils, polyphenols, and other bioactive compounds^23^.

The reported anti-inflammatory potential of *Lavandula* species and their constituents prompted us to investigate the anti-inflammatory activity of the compounds isolated from *L. coronopifolia*. A scratch wound assay using J774A.1 macrophage-like cells stimulated with lipopolysaccharide (LPS) from *Escherichia coli*, a known pro-inflammatory agent^26^, was implemented. This assay was used as an indirect approach to assess inflammation by evaluating changes in cell migration in the presence of the isolated compounds. J774A.1 macrophages, a murine monocyte cell line, are extensively employed as an in vitro model to investigate the behavior of macrophages under inflammatory conditions, including migration and functional responses. Upon stimulation with proinflammatory agents such as LPS, monocyte cells exhibit upregulated expression of cytokines such as tumor necrosis factor- α (TNF-α), interleukin-1β (IL-1β), and interleukin 6 (IL-6) accompanied by enhanced migratory activity^27–30^. Anti-inflammatory agents can effectively attenuate or suppress these proinflammatory signaling cascades, thereby restoring cellular homeostasis and limiting tissue damage^31,32^. The scratch wound assay was conducted to assess the migratory capacity of the cells under LPS-induced inflammatory conditions^33,34^. Therefore, this technique was used as an indirect measure of inflammation by evaluating changes in cell migration. Dexamethasone was used as a positive control, while PBS served as a negative control.

**Fig. 3:**
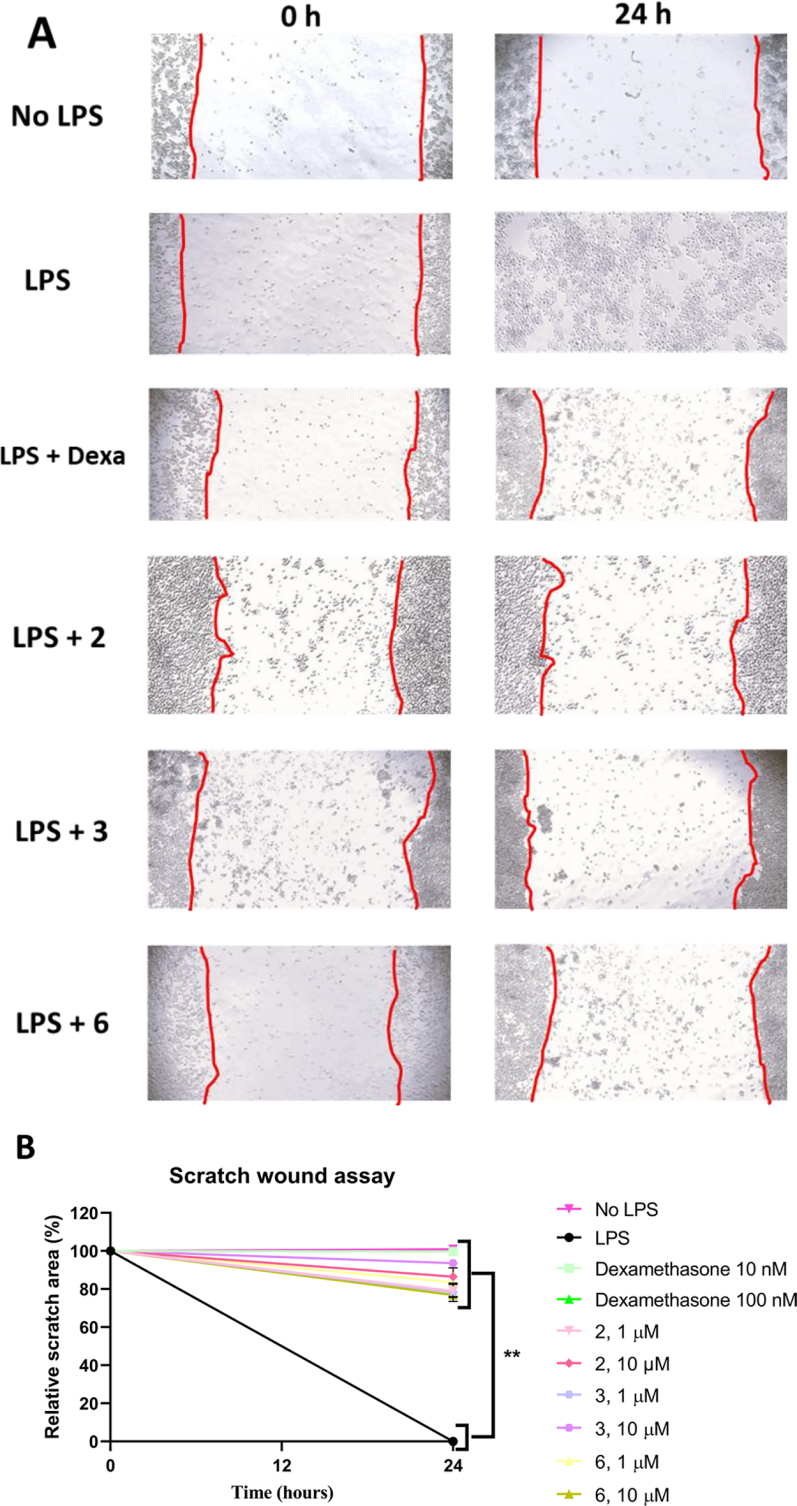
Scratch wound assay. J774A.1 cells were treated with 1 or 10 µM of compounds **2**, **3**, and **6**, using dexamethasone (10 and 100 nM) as a positive anti-inflammatory control and PBS as vehicle. (**A**) Representative images (10x objective) were taken at 0 and 24 h post-treatment. (**B**) Relative scratch area was calculated as the percentage of the scratch area remaining open after 24 h, and the results were normalised to both controls. Data are expressed as mean ± SD from two independent experiments, each performed in duplicate. Statistical differences were evaluated using one-way ANOVA. Significance levels are indicated as ** at *p* < 0.01 compared with LPS.

First, the migration capacity of macrophages in the presence or absence of LPS was assessed to confirm that LPS stimulation enhances macrophage migration in 2D models. Untreated J774A.1 cells exhibited negligible migration, whereas LPS treatment markedly enhanced their motility, leading to almost full scratch closure within 24 h (Fig. 3).

To determine the nontoxic anti-inflammatory concentrations, each compound was firstly screened at the highest concentrations that are compatible with its cytotoxicity profile in HEK293T (Figure S29). Caffeic acid **1** showed moderate activity at 2.5 µM but no effect at 5 µM, possibly due to cytotoxicity at higher concentrations that may exacerbate the inflammatory response due to cell damage^35^. Methyl rosmarinate **2** and the ursane derivatives **3** and **6** displayed the most promising anti-inflammatory profiles.

### Compounds **2**, **3** and **6** suppressed cell migration

The best-performing anti-inflammatory candidates **2**, **3**, and **6** were subsequently re-tested at lower concentrations (1 and 10 µM) to confirm their activity (Fig. 3). Following wound induction, representative images were taken for each treatment (Fig. 3A), and the area normalized percentage for each compound was assessed after 24 h relative to time 0. In the negative control group, treatment of J774A.1 cells with LPS agent markedly promoted cell migration, resulting in an approximately 90% reduction of the scratched area. In contrast, dexamethasone-treated cells (positive control) exhibited a pronounced inhibition of LPS-induced migration, with the scratched area reduced only by ~ 10%.

After 24 h of treatment, all tested compounds significantly limited the reduction in the scratched area, indicating a suppression of cell migration. Compounds **2** and **3** exhibited strong inhibitory effects on cell migration at both tested concentrations, where they reduced the scratch area by 13.5 and 6.4% at 10 µM; and by 20.9 and 21.7% at 1 µM, respectively. These results indicate that both compounds retained substantial anti-migratory potency at low concentrations.

Compound **6** exhibited comparable effects at both concentrations tested, showing 19.6 and 23.1% reductions in the normalized scratch area at 1 µM and 10 µM, respectively. These differences were not statistically significant between the two concentrations (Fig. 3B), suggesting strong anti-inflammatory and anti-migratory activities of **6**.

### Compounds **2**, **3** and **6** affected macrophage morphology after LPS activation

**Fig. 4:**
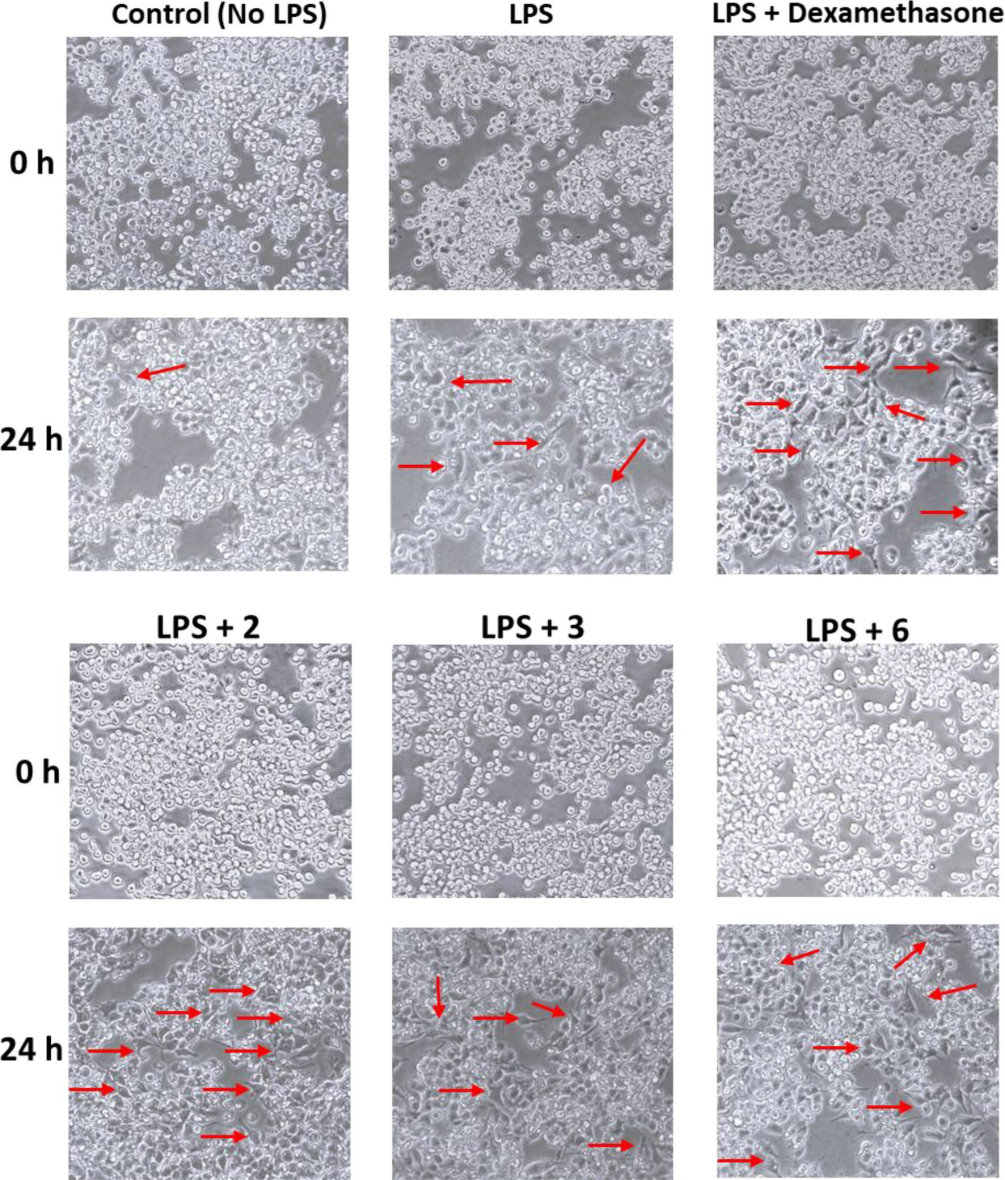
J774A.1 cell morphology analysis after LPS activation in absence of tested compounds (untreated) or treated with dexamethasone (10 nM), or different tested compounds **2**, **3** and **6** (10 µM) compared with non-induced cells (without LPS), for 24 h. The red arrows highlight areas where there are cell clusters with different shapes. The figure shows representative images from two independent biological replicates; each performed in duplicate.

Changes in cell morphology can indirectly reflect both cell migration and the action of anti-inflammatory drugs, which are reported to influence cell shape and structure^36,37^. Therefore, the effect of LPS activation on cell morphology, in addition to the effects of compounds **2**, **3** and **6** on cell shape, and differentiation enhancement were tested (Fig. 4). In the absence of LPS, the cells retained their characteristic rounded appearance and displayed high confluence. The addition of LPS promoted a slightl morphological change in the cells compared to the control without LPS activation. However, consistent with previous reports, treatment with dexamethasone markedly altered cell differentiation and morphology, resulting in a more pronounced spindle-shaped appearance^37^. Similar results were observed for compounds **2**, **3**, and **6**, supporting their anti-inflammatory action. This morphological differentiation is driven by the proliferative response induced by LPS, which promotes continued cell growth and activation. In contrast, the addition of anti-inflammatory agents reduces cell proliferation and motility, leading to increased cell attachment and a more differentiated morphology^38^.

### Compounds **2**, **3** and **6** reduced inflammation through iNOS and IL-6 downregulation

Inflammation is a complex cellular response that can be elicited by different mechanisms, including Damage-Associated Molecular Patterns (DAMPs), Resolution-Associated Molecular Patterns (RAMPs) or Specialized Pro-Resolving Mediators (SPMs), among others^34,35^. Inflammation induced by LPS is typically associated with the activation of Toll-like receptor 4 (TLR4), which triggers the expression of pro-inflammatory cytokines such as IL-6 through the NF-κB pathway^29^. Additionally, LPS-induced cell motility is commonly linked to the Src family kinases (SFKs) and Focal Adhesion Kinase (FAK), referred to as SFK-FAK cascade**,** which is associated with inducible nitric oxide synthase (iNOS) activity; this enzyme is upregulated in defense cells during inflammatory processes^39^.

Based on this rationale, the expression of IL-6, and iNOS inflammation-related biomarkers were quantified by qPCR after 24 h stimulation of J774A.1 cells with bacterial LPS (1 µg/mL). Because the expression of these inflammation-related factors varies with exposure time, we selected a 24 h incubation with the test compounds in the presence of LPS to assess the inflammatory status, following the same conditions used in previous experiments to ensure consistency and enable direct comparison of the results.

**Fig. 5:**
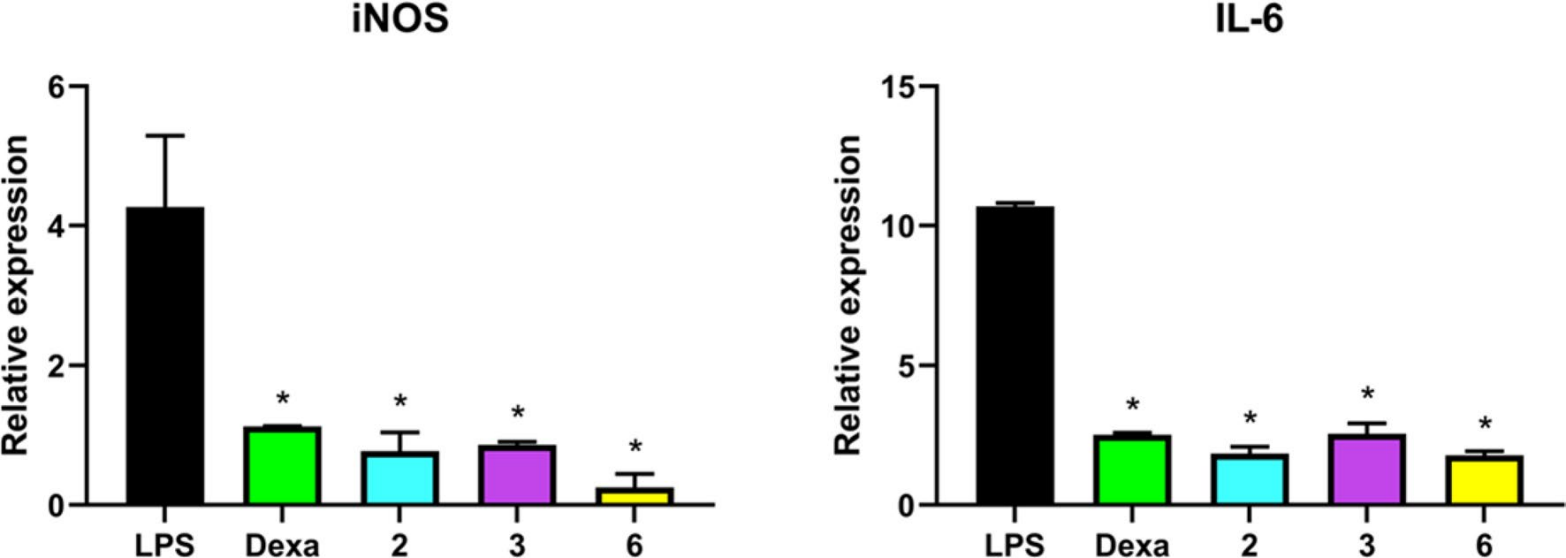
qPCR analysis of IL-6 and iNOS in J774A.1 macrophages activated with LPS (1 µg/mL). The results represent the 2^-ΔΔCt^ for two biological replicates with two replicates technical each. The results were normalized against actin, and cells non activated with LPS were used as the control. Statistical differences were evaluated using t-test. Data represents mean ± SD from two independent biological replicates, each performed with two technical replicates. Significance levels are indicated as * at *p* < 0.05.

After the 24 h exposure, the expression of IL-6, and iNOS was evaluated (Fig. 5). The results clearly show that all tested compounds downregulated iNOS expression (relative expression values < 1) and significantly reduced the LPS-induced overexpression of IL-6.

Downregulation of iNOS represents a highly significant anti-inflammatory effect, as the link between iNOS expression and inflammation is well documented in macrophages treated with LPS^39^. The decrease in iNOS expression is expected to correlate with a reduction in nitric oxide (NO) production, which constitutes the classical response of macrophages following LPS activation. Moreover, NO production, a direct byproduct of iNOS activity, has been associated with cell motility through the SFK–FAK pathway.

Given the complexity of inflammatory signaling, we focused our analysis on the expression of key pro-inflammatory markers associated with LPS activation, especially IL-6 and iNOS which have been widely reported in LPS-stimulated macrophages^40–43^. Accordingly, we evaluated IL-6 expression levels at 24 h post-LPS induction. IL-6 expression was significantly reduced from that of the LPS-induced control. The tested compounds attenuated IL-6 overexpression in a manner comparable to dexamethasone, which has also been previously associated with IL-6 downregulation^29^. In fact, the relative IL-6 expression values were similar to those obtained in the non-stimulated control (the reference condition used for the 2^−ΔΔCt^ calculation), indicating that the compounds effectively reduced the inflammatory response to levels comparable to non-activated macrophages.

The findings of this study are in agreement with the reported anti-inflammatory activity of ursane derivatives. Ursane-type triterpenoids, a class of pentacyclic compounds found in many plants, have attracted significant attention for their anti-inflammatory properties. Ursane-derivatives are reported to exert their anti-inflammatory effects through multiple mechanisms including the suppression of NO production, and the reduction of pro-inflammatory cytokines like IL-6 in activated macrophages^44,45^, These effects are thought to involve the modulation of key signaling pathways central to inflammation, including nuclear factor kappa B (NF-κB), nuclear factor of activated T cells (NF-AT), mitogen-activated protein kinase (MAPK), and activator protein-1 (AP-1)^46–48^. However, it should be noted that the involvement of these pathways was not experimentally investigated in the present study. Furthermore, methyl rosmarinate (**2**) is reported to inhibit the LPS-induced expression of pro-inflammatory cytokines including IL-6 and the production of NO through suppression of NF-κB signaling pathway^49^. Another study reported it inhibited IL-6 and iNOS in the LPS-RAW 264.7^50^.

## Conclusion

In the current study, two phenylpropanoids (**1**–**2**) and five ursane triterpenes were isolated from *L. coronopifolia*. 2α, 3*β*, 23-Trihydroxyurs-12,18-dien-28-oic acid 28-*O-β*-d-glucopyranoside (**5**) showed anti-lung cancer activity with a good therapeutic index. The anti-inflammatory potential of the compounds was assessed in LPS-stimulated macrophage cells. Methyl rosmarinate (**2**), 1*β*, 2*α*, 3*β*, 19*α*, 23-pentahydroxy-urs-12-en-28-oic acid-28-*O*-*β*-d-glucopyranoside (**3**) and 2*α*, 3*β*, 23-trihydroxyurs-12, 19-dien-28-oic acid 28-*O*-*β*-d-glucopyranoside (**6**) attenuated LPS-induced migration and morphological changes. In addition, **2**, **3** and **6** significantly reduced the expression levels of the inflammatory mediators, iNOS and IL-6. The data support the hypothesis about the inhibitory effects of these compounds on pathways classically associated with LPS responses, such as NF-κB, although this was not directly tested in this study. The findings provide a molecular basis to understand the anti-inflammatory activity of *L. coronopifolia*. This study also underscores the significance of ursane-type triterpenoids as promising anticancer and anti-inflammatory agents and demonstrates how subtle structural modifications can markedly influence their biological activities. The presence of a hydroxyl group at C-1 of the ursane nucleus appears to be important for reducing toxicity towards non tumoral control cell line HEK293T cells and for enhancing anti-inflammatory activity, as observed with compound **3**. Furthermore, the presence and position of a double bond in ring E play a key role in determining bioactivity: a double bond between C-18 and C-19 may be critical for anticancer activity (compound **5**), whereas a double bond between C-19 and C-20 may favour anti-inflammatory activity (compound **6**).

## Methods

### General

Thin-layer chromatography was carried out using Merck precoated silica gel F254 plates (E-Merck, Germany) and using vanillin–sulfuric acid spray reagent. Colum Chromatography was carried out using Silica gel G 60–230 (Merck, Germany), C-18 reversed silica gel 38–63 μm (Wakogel 50C18, Wako Pure Chemical Industries, Ltd., Osaka, Japan) and Sephadex LH-20 (GE Healthcare Bio-sciences AB, Sweden). The solvents, including *n*-hexane, methylene chloride (CH_2_Cl_2_), ethyl acetate (EtOAc) and *n*-butanol (*n*-BuOH), were of reagent grade (El-Nasr Co., Abu Zaabal – Kalyoubia, Cairo, Egypt). Nuclear Magnetic Resonance spectra measurements were carried out on Bruker Avance III HD-400 spectrometer at 400 MHz for ^1^H and 100 MHz for APT in NMR unit, Faculty of Pharmacy, Mansoura, Egypt. Chemical shifts (δ) are expressed in ppm with reference to the residual solvent signal. Coupling constants (*J* values) are given in Hz. LC‑MS spectra of compounds **1**–**7** were acquired on an Orbitrap IQ‑X MS coupled to a Vanquish HPLC system (both Thermo Scientific, Frankfurt, Germany). The mobile phase consisted of solvent A (0.1% formic acid in water) and solvent B (0.1% formic acid in 80% acetonitrile in water). A Phenomenex Kinetex EVO C18 column (100 × 2.1 mm, 2.6 µm) was used with linear gradient from 5.0% to 85% of solvent B over 18 min at a constant flow rate of 0.3 mL/min. Compounds were ionized using heated electrospray ionization (HESI) in negative ion mode. The Orbitrap operated with the following parameters: auxiliary gas flow 10 Arb, sheath gas flow 50 Arb, sweep gas 1 Arb, ion transfer tube temperature 275 °C, vaporizer temperature 350 °C, and spray voltage + 3.5 kV. Lock mass correction was performed using EASY-IC. Full MS scans were collected over *m/z* range of 150–2000 at a resolution of 120,000, with an RF lens of 35% and a normalized AGC target of 100% (centroid mode). Raw data were processed using Thermo Freestyle software.

### Plant material

The plant material consisted of the flowering aerial parts of *Lavandula coronopifolia* Poir. (Lamiaceae). It was collected from Suez, Wady Hagool in Feburary 2017 and authenticated by Prof. Ibrahim Mashaly, at Ecology and Botany Department, Faculty of Science, Mansoura University. A voucher specimen has been deposited at the herbarium of the Pharmacognosy Department, Faculty of Pharmacy, Mansoura University (02–17-LC-Mansoura).

### Extraction and isolation

Two and a half Kilograms of the dried powdered aerial parts of *L. coronopifolia* were extracted exhaustively using 90% MeOH to give 510 g total extract. The dried extract was dissolved in MeOH/H_2_O (50/50) and then extracted successively with *n*-hexane, CH_2_Cl_2_, EtOAc and finally with *n*-butanol in a separating funnel. The solvents, in each case, were distilled to dryness under reduced pressure to afford 34.1 g, 17.5 g, 46.8 g and 25 g of *n*-hexane, CH_2_Cl_2_, EtOAc and *n*-butanol extracts, respectively. The detailed isolation procedures for **1**–7 are available in the supporting file.

### Cytotoxicity assay

Human lung adenocarcinoma cells (A549, CCL-185) and human embryonic kidney cells (HEK293T, CRL-3216) were obtained from ATCC (American Type Culture Collection). They were used to evaluate the cytotoxicity of the pure compounds isolated from *L. coronopifolia*. The cells were cultured in RPMI medium supplemented with 10% fetal bovine serum (FBS) at 37 °C in a humidified atmosphere containing 5% CO₂. Cell viability was assessed using standard MTT procedures. Briefly, 5 × 10^4^ cells were seeded in 100 µL of medium in 96-well plates and cultured overnight. After 24 h, the cells were washed once with pre-warmed Dulbecco’s phosphate-buffered saline (DPBS), and the medium was replaced with serum-free RPMI. The isolated compounds were prepared at various concentrations, and 40 µL of each concentration was added to the corresponding wells. The cells were incubated for 2 h, after which 10% FBS was added to each well. The plates were further incubated at 37 °C in a humidified atmosphere containing 5% CO₂ for 48 h. Following incubation, 20 µL of MTT solution (3-(4,5-dimethylthiazol-2-yl)-2,5-diphenyltetrazolium bromide, 5 mg/mL) was added to each well and incubated for 3 h. Thereafter, the medium was discarded, and 100 µL of dimethyl sulfoxide (DMSO) was added per well to solubilize the formazan crystals. The plates were incubated at 37 °C for 10 min with gentle shaking. Absorbance was measured at 595 nm using a Victor NIVO microplate reader. Final values were analyzed by fitting the data to a log inhibitor versus response (three-parameter) equation using Prism (GraphPad Software, San Diego, CA, USA). The experiment was performed using three independent biological replicates, each analyzed in two technical replicates.

### Scratch wound assay

The scratch wound assay was performed following previously described protocols^51^ with minor modifications. J774A.1 cells WT (RRID: CVCL_0358) were obtained from ATCC. The cells (3 × 10^5^ cells/well) were seeded into six-well cell culture plates. After reaching approximately 80% confluence, the medium was replaced with serum-free RPMI and cells were incubated for 12 h. A scratch was then made across the monolayer using a sterile pipette tip, and the medium was replaced with RPMI containing 1 µg/mL lipopolysaccharide (LPS), to induce inflammation. Phosphate-buffered saline (PBS) was used as a negative control and dexamethasone as a positive anti-inflammatory control. The tested compounds were added at various concentrations. Images were captured at 0 and 24 h using an inverted microscope (Motic ae2000), and wound areas were quantified using ImageJ software. The experiment was performed using three independent biological replicates, each analyzed in two technical replicates.

### RNA isolation, RT-qPCR analysis and cell morphology examination

To measure the levels of inflammation biomarkers, we seeded the cells into 6-well plates and treated them as previously described for the scratch wound assay. Once the cells reached confluency and treated with LPS, an initial picture was taken using an inverted Motic AE2000 microscope with a 10× objective. After 24 h of incubation with the tested compounds and controls, we took another picture before proceeding with RNA purification.

J774A.1 cells were harvested by scraping in cold PBS, followed by centrifugation at 3,000 × g for 5 min. After removing the supernatant, the cell pellet was stored at − 80 °C. Total RNA was extracted using the RNeasy Mini Kit (Qiagen, Spain) according to the manufacturer’s instructions. RNA concentration and purity were assessed using a NanoDrop™ 2000 spectrophotometer (Thermo Fisher Scientific™, Spain) before RNA reverse transcription.

Reverse transcription qPCR (RT-qPCR) was performed using the One-Step TB Green PrimeScript RT-qPCR Kit II (Takara Bio, Spain) on a QuantStudio 5 system (Applied Biosystems, USA). Primer sequences were obtained from Yamakawa et al.^52^. Each reaction was carried out in a final volume of 20 µL, following the manufacturer’s recommendations. RNA samples were normalized to 50 ng/µL prior to being added to the reaction mixture. The thermocycling program consisted of an initial reverse transcription step at 42 °C for 5 min, followed by denaturation at 95 °C for 10 s, and 40 amplification cycles (95 °C for 5 s and 60 °C for 30 s).

Relative gene expression levels were calculated using the 2^−ΔΔCt^ method, using β-actin as the endogenous control and non–LPS-treated cells as the reference condition for relative expression. Fold-change values are shown in the figures, while statistical analyses were performed using the corresponding ΔCt values. The experiment was repeated with two biological replicates and two technical replicates of each.

### Statistical analysis

Statistical analyses were performed using GraphPad Prism (version 8.0.1; GraphPad Software, San Diego, CA, USA). Normality and homoscedasticity were verified prior to conducting one-way ANOVA, followed by Tukey´s multiple comparison post-hoc test, with statistical significance set at *p* ≤ 0.05.

## Supplementary Information

Supplementary Information.

## Data Availability

The datasets used in this study are included in this article and its supplementary information files.
