# Supplementary material for: Attenuation of LPS-induced inflammatory responses in J774A.1 macrophages by phenylpropanoids and ursane triterpenes from Lavandula coronopifolia Poir
Source: Sci Rep. 2026 May 24;16:16021. doi: 10.1038/s41598-026-51849-5 (PMC13199454; doi:10.1038/s41598-026-51849-5)

**Attenuation of LPS-induced inflammatory responses in J774A.1 macrophages by phenylpropanoids and ursane triterpenes from *Lavandula coronopifolia* Poir.**

Marwa Elsbaey^a*^, Eman Elattar^a^, Álvaro Mourenza^b^, Pablo Castañera^b^, Luis M. Mateos^b^, Michal Letek^b^, Mai H. ElNaggar^c†^

^a^ Department of Pharmacognosy, Faculty of Pharmacy, Mansoura University, Mansoura 35516, Egypt

^b^ Departamento de Biología Molecular, Área de Microbiología, Universidad de León, 24071 León, Spain

^c^Department of Pharmacognosy, Faculty of Pharmacy, Kafrelsheikh University, 33511, Kafrelsheikh, Egypt

***Corresponding author:** Marwa Elsbaey,

Email: [marwaelsebay1611@mans.edu.eg](mailto:marwaelsebay1611@mans.edu.eg), Tel.: 2001066364811; Fax: +02(050)22005520,

Postal address: Department of Pharmacognosy, Faculty of Pharmacy, Mansoura University, 25 El Gomhouria St, Dakahlia Governorate 35516-Mansoura- Egypt

^†^**Co-corresponding author:** Mai H. ElNaggar

**Email address:** [mai_elnaggar@pharm.kfs.edu.eg](mailto:galal_magdy@pharm.kfs.edu.eg)

**Table of Contents**

| **Content** | **Page** |
| --- | --- |
| **Figure S1.** ^1^H NMR spectrum of **1** (400 MHz, CD_3_OD). | 4 |
| **Figure S2.** APT spectrum of **1** (100 MHz, CD_3_OD). | 5 |
| **Figure S3.** LC-MS spectrum of **1** in negative ion mode. | 6 |
| **Figure S4.** ^1^H NMR spectrum of **2** (400 MHz, CD_3_OD). | 7 |
| **Figure S5.** APT spectrum of **2** (100 MHz, CD_3_OD). | 8 |
| **Figure S6.** LC-MS spectrum of **2** in negative ion mode. | 9 |
| **Figure S7.** ^1^H NMR spectrum of **3** (400 MHz, CD_3_OD). | 10 |
| **Figure S8.** APT spectrum of **3** (100 MHz, CD_3_OD). | 11 |
| **Figure S9.** LC-MS spectrum of **3** in negative ion mode. | 12 |
| **Figure S10.** ^1^H NMR spectrum of **4** (400 MHz, CD_3_OD). | 13 |
| **Figure S11.** APT spectrum of **4** (100 MHz, CD_3_OD). | 14 |
| **Figure S12.** LC-MS spectrum of **4** in negative ion mode. | 15 |
| **Figure S13.** ^1^H NMR spectrum of **5** (400 MHz, CD_3_OD). | 16 |
| **Figure S14.** APT spectrum of **5** (100 MHz, CD_3_OD). | 17 |
| **Figure S15.** LC-MS spectrum of **5** in negative ion mode. | 18 |
| **Figure S16.** ^1^H NMR spectrum of **6** (400 MHz, CD_3_OD). | 19 |
| **Figure S17.** APT spectrum of **6** (100 MHz, CD_3_OD). | 20 |
| **Figure S18.** LC-MS spectrum of **6** in negative ion mode. | 21 |
| **Figure S19.** ^1^H NMR spectrum of **7** (400 MHz, CD_3_OD). | 22 |
| **Figure S20.** APT spectrum of **7** (100 MHz, CD_3_OD). | 23 |
| **Figure S21.** LC-MS spectrum of **7** in negative ion mode. | 24 |
| **Figure S22.** ^1^H NMR spectrum of chromatographic fractions 16-21 (400 MHz, CD_3_OD) prior HPLC purification. The mixture contains compounds **5**, **6** and **7**. | 25 |
| **Figure S23.** APT spectrum of chromatographic fractions 16-21 prior HPLC purification (100 MHz, CD_3_OD). The mixture contains compounds **5**, **6** and **7**. | 26 |
| **Figure S24.** Stacked APT spectra (100 MHz, CD3OD) comparing chromatographic fractions 16-21 with compounds **6** and **7**, expanded at δ 110-155 ppm. Key diagnostic carbons for compound **5** are indicated. | 27 |
| **Figure S25.** Stacked APT spectra (100 MHz, CD_3_OD) comparing chromatographic fractions 16-21 with compounds **6** and **7**, expanded at δ 13-30 ppm. | 28 |
| **Figure S26.** Stacked APT spectra (100 MHz, CD_3_OD) comparing chromatographic fractions 16-21 with compounds **6** and **7**, expanded at δ 31-48 ppm. | 29 |
| **Figure S27.** Stacked APT spectra (100 MHz, CD_3_OD) comparing chromatographic fractions 16-21 with compounds **6** and **7**, expanded at δ 51-79 ppm. | 30 |
| **Figure S28.** Stacked APT spectra (100 MHz, CD_3_OD) comparing chromatographic fractions 16-21 with compounds **6** and **7**, expanded at δ 176-178 ppm. | 31 |
| **Figure S29.** Scratch wound assay. J774.1 cells were treated with the highest concentrations compatible with their cytotoxicity profiles, using dexamethasone (100 nM) as a positive anti-inflammatory control and PBS as vehicle. | 32 |
| Chromatographic isolation of compounds **1**-**7** | 33 |
| **Figure S30.**  HPLC chromatogram of compounds **5**-**7** before separation detected at 200 nm. | 34 |
| **Figure S31.**  Thinn-layer chromatography (TLC) chromatogram (Silica gel GF_254_, using EtOAc-MeOH-H_2_O, 9:0.5:0.5 *v*/v as the solvent system) of compounds **5**-**7** after purification; **A:** Visualization under UV light at 254 nm shows quenching only for compound **5**, consistent with the presence of a conjugated double bond; **B:** Visualization using vanillin-sulfuric spray reagent and heating. | 36 |
| **Figure S32.**  HPLC-RP chromatograms for compounds **5**-**7** after purification detected at 200 nm using the same conditions; **A.** compound **5** eluted at min. 13.739; **B.** compound **6** eluted at min. 13.849; **C.** compound **7** eluted at min. 13.983. | 37 |
| **Figure S33.**  HPLC-RP chromatograms for compounds **5**-**7** after purification detected at 210 nm using the same conditions; **A.** compound **5** eluted at min. 13.739; **B.** compound **6** eluted at min. 13.849; **C.** compound **7** eluted at min. 13.983. | 38 |

**Figure S1.** ^1^H NMR spectrum of **1** (400 MHz, CD_3_OD).

**Figure S2.** APT spectrum of **1** (100 MHz, CD_3_OD).

**Figure S3.** LC-MS spectrum of **1** in negative ion mode.

**Figure S4.** ^1^H NMR spectrum of **2** (400 MHz, CD_3_OD).

**Figure S5.** APT spectrum of **2** (100 MHz, CD_3_OD).

**Figure S6.** LC-MS spectrum of **2** in negative ion mode.

**Figure S7.** ^1^H NMR spectrum of **3** (400 MHz, CD_3_OD).

**Figure S8.** APT spectrum of **3** (100 MHz, CD_3_OD).

**Figure S9.** LC-MS spectrum of **3** in negative ion mode.

Formic acid adduct

[M + 46.00495]^-^

**Figure S10.** ^1^H NMR spectrum of **4** (400 MHz, CD_3_OD).

**Figure S11.** APT spectrum of **4** (100 MHz, CD_3_OD).

**Figure S12.** LC-MS spectrum of **4** in negative ion mode.

Formic acid adduct

[M + 46.00484]^-^

**Figure S13.** ^1^H NMR spectrum of **5** (400 MHz, CD_3_OD).

**Figure S14.** APT spectrum of **5** (100 MHz, CD_3_OD).

**Figure S15.** LC-MS spectrum of **5** in negative ion mode.

Formic acid adduct

[M + 46.00436]^-^

**Figure S16.** ^1^H NMR spectrum of **6** (400 MHz, CD_3_OD).

**Figure S17.** APT spectrum of **6** (100 MHz, CD_3_OD).

**Figure S18.** LC-MS spectrum of **6** in negative ion mode.

Formic acid adduct

[M + 46.0067]^-^

**Figure S19.** ^1^H NMR spectrum of **7** (400 MHz, CD_3_OD).

**Figure S20.** APT spectrum of **7** (100 MHz, CD_3_OD).

**Figure S21.** LC-MS spectrum of **7** in negative ion mode.

Formic acid adduct

[M + 46.0065]^-^

**Figure S22.** ^1^H NMR spectrum of chromatographic fractions 16-21 (400 MHz, CD_3_OD) prior HPLC purification. The mixture contains compounds **5**, **6** and **7**.

**Figure S23.** APT spectrum of chromatographic fractions 16-21 prior HPLC purification (100 MHz, CD_3_OD). The mixture contains compounds **5**, **6** and **7**.

**Figure S24.** Stacked APT spectra (100 MHz, CD_3_OD) comparing chromatographic fractions 16-21 with pure compounds **6** and **7**, expanded at δ 110-156 ppm. Key diagnostic carbons for compound **5** are indicated.

**Fractions 16-21**

**(Compounds 5, 6 and 7)**

**Compound 7**

**Compound 7**

**Compound 6**

**Figure S25.** Stacked APT spectra (100 MHz, CD_3_OD) comparing chromatographic fractions 16-21 with pure compounds **6** and **7**, expanded at δ 13-30 ppm.

**Fractions 16-21**

**(Compounds 5, 6 and 7)**

**Compound 7**

**Compound 6**

**Figure S26.** Stacked APT spectra (100 MHz, CD_3_OD) comparing chromatographic fractions 16-21 with compounds **6** and **7**, expanded at δ 31-48 ppm.

**Fractions 16-21**

**(Compounds 5, 6 and 7)**

**Compound 7**

**Compound 6**

**Figure S27.** Stacked APT spectra (100 MHz, CD_3_OD) comparing chromatographic fractions 16-21 with compounds **6** and **7**, expanded at δ 51-79 ppm.

**Fractions 16-21**

**(Compounds 5, 6 and 7)**

**Compound 7**

**Compound 6**

**Figure S28.** Stacked APT spectra (100 MHz, CD_3_OD) comparing chromatographic fractions 16-21 with compounds **6** and **7**, expanded at δ 176-178 ppm.

**Fractions 16-21**

**(Compounds 5, 6 and 7)**

**Compound 7**

**Compound 6**

**Figure S29.**  Scratch wound assay. J774.1 cells were treated with the highest concentration of each compound compatible with their cytotoxicity profiles, using dexamethasone (100 nM) as a positive anti-inflammatory control and PBS as vehicle.


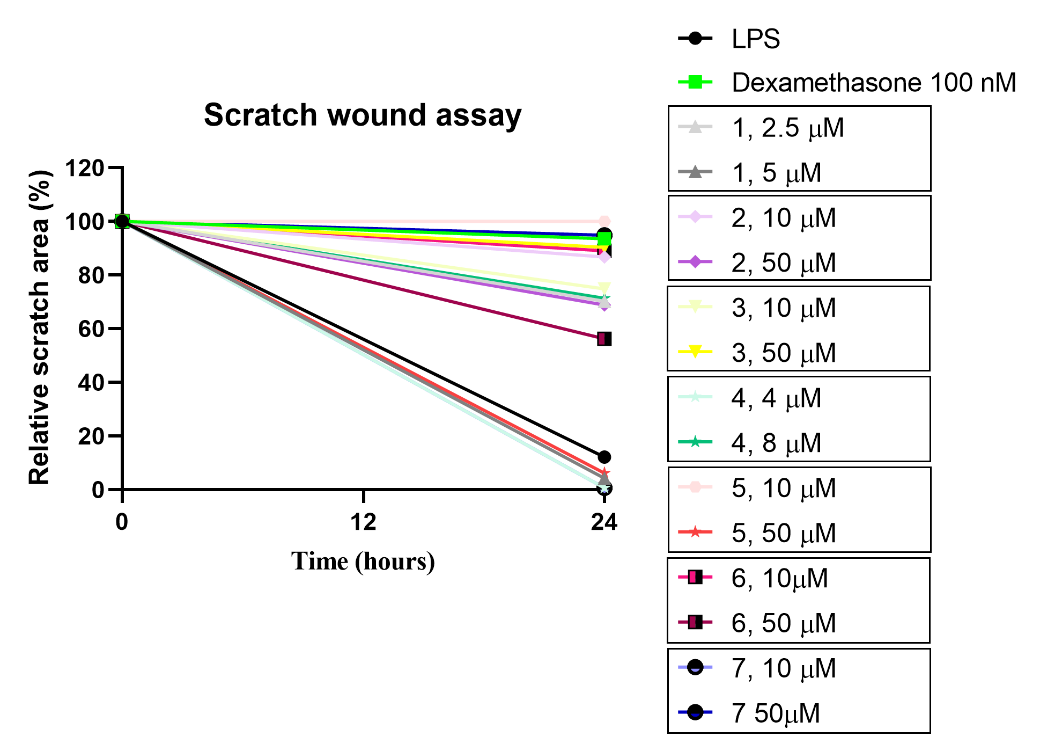


**Chromatographic isolation of compounds 1-7**

About 45 grams of the EtOAc extract was subjected to coarse fractionation using normal-phase column chromatography (25 cm internal diameter x 8 cm length). The column was packed with 350 g of silica gel for adopting a gradient elution method with EtOAc in petroleum ether (from 20 to 100%), followed by MeOH in EtOAc (from 0 to 40%). Fractions of 200 mL were collected, concentrated, and screened by TLC. Based on TLC screening, similar fractions were pooled together to provide seven groups (A-G).

**Group C (Fr. 13-19, 3.3 g)**, eluted with 50-60% EtOAc in petroleum ether, was subjected to reversed-phase column chromatography (25 x 1 cm) using gradient elution of MeOH/H_2_O, 10 mL fractions were collected. Fractions 3-6, eluted with 40% MeOH in H_2_O, afforded **1** (39.8 mg) as needle shaped crystals. Fractions 8-42 (1.476 g), eluted with 30-40% MeOH in H_2_O, were pooled together and further subjected to normal-phase silica gel chromatography (25 x 1.5 cm) using CH_2_Cl_2_/MeOH as an eluting solvent. Fractions 52-72, eluted with 3% MeOH in CH_2_Cl_2,_ afforded **2** (275 mg) as brown amorphous material.

**Group F (Fr. 35-46, 15.9 g)**, eluted with 5-20% MeOH in EtOAc, was subjected to normal-phase vacuum liquid chromatography (VLC, ϕ 6 cm internal diameter × 8 cm length). It was packed with 150 g of silica gel in EtOAc (100%), adopting a gradient elution method with EtOAc in MeOH. 200 mL fractions were collected, concentrated, and screened by TLC. Fractions (3-4, 3.3g) eluted with 10-20% MeOH was chromatographed over Sephadex LH20 (50 g, 2 cm internal diameter) using isocratic elution with CH_2_Cl_2_/MeOH (1/1), 20 mL fractions were collected. Fractions (13-24) were pooled together and subjected to normal-phase silica gel column (28 X 2 cm) using gradient elution of MeOH in EtOAc (from 0 to 50%), and 50 mL fractions were collected. Fractions 18-23, 375 mg, eluted with 9-12% MeOH in EtOAc were subjected to reversed-phase column chromatography (32 x 1.5 cm) using isocratic elution of MeOH/H_2_O (45/65) and 10 mL fractions were collected. Fractions 26-33 afforded white precipitate coded as **3** (45.6 mg). Fractions 43-46 afforded white precipitate coded as **4** (27.9 mg). Fractions 60-65 were eluted with 100% MeOH. They were further purified over Sephadex LH20 (40 x 2 cm, 65 g) using 100% MeOH, 2 mL fractions were collected, to afford fractions 2-3. Fractions 2-3 were further subjected to a normal-phase silica gel VLC (8 x 2 cm), adopting gradient elution of MeOH in CH_2_Cl_2_, 2 mL fractions were collected, concentrated, and screened by TLC. NMR analysis of fractions 16-21 (7 mg, eluted with 7.5 % MeOH) showed the presence of three triterpenes (Figures S15-S21). Accordingly, they were further subjected to purification using a Dionex UltiMate™ 3000 analytical reversed-phase HPLC system (Thermo Scientific™, Dionex™, Sunnyvale, CA, USA), equipped with an LPG-3400SD quaternary pump, a WPS-3000TSL autosampler, a TCC-3000SD column thermostat, and a DAD-3000 diode array detector, controlled by Chromeleon Chromatography Studio software. The chromatographic separation was performed on a BDS Hypersil™ C18 column (250 × 4.6 mm, 5 µm particle size) by making about 100 injections (each of 20 µL) of the mixture under purification and gradient elution with 30.0-30.2 % acetonitrile in water system over 30 min. The flow rate was adjusted to 0.5 mL/min. The eluted compounds were collected manually based on the DAD-3000 diode array detector signal (Figure S30) to afford three compounds coded as **5** (2.4 mg), **6** (1.4 mg), and **7** (2.0 mg).

The purity of the separated compounds was assessed by silica gel GF_254_ thin-layer chromatography (TLC) using EtOAc-MeOH-H_2_O, 9:0.5:0.5 *v*/v as the solvent system. The visualized chromatogram under UV light at 254 nm showed quenching only for compound **5**, consistent with the presence of a conjugated double bond. While visualization using vanillin-sulfuric spray reagent and heating showed purple-colored spots for the 3 separated compounds (Figure S31). Purity was further assessed by reanalysis of the purified compounds following separation using an Agilent 1260 Infinity II HPLC system equipped with a G7112B binary pump, a G7129A autosampler, and a G7115A diode array detector, controlled by OpenLAB software. Samples were prepared at approximately 0.1 mg/100 µL, and 30 µL was injected. Chromatographic analysis was performed on an Agilent Zorbax SB‑C18 column (250 × 4.6 mm, 5 µm particle size), using a gradient elution of 5.0–100% acetonitrile in water over 30 min at a flow rate of 1 mL/min. The HPLC-RP chromatograms revealed single, pure peaks for compounds **5**, **6**, and **7**, with retention times of 13.739, 13.849, and 13.983 min, respectively. The peaks were detected at both 200 nm (Figure S32) and 210 nm (Figure S33).

**Figure S30.**  HPLC-RP chromatogram of compounds **5**-**7** before separation detected at 200 nm.


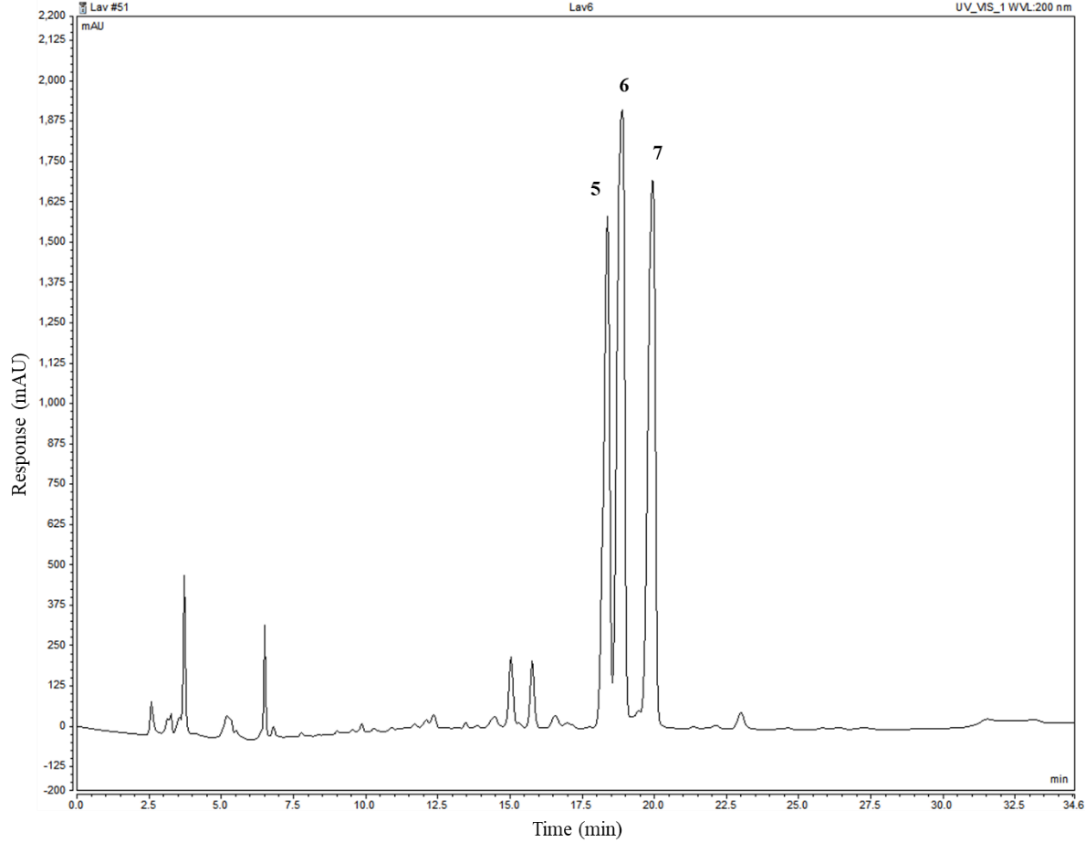


**Figure S31.**  Thinn-layer chromatography (TLC) chromatogram (Silica gel GF_254_, using EtOAc-MeOH-H_2_O, 9:0.5:0.5 *v*/v as the solvent system) of compounds **5**-**7** after purification; **A:** Visualization under UV light at 254 nm shows quenching only for compound **5**, consistent with the presence of a conjugated double bond; **B:** Visualization using vanillin-sulfuric spray reagent and heating.


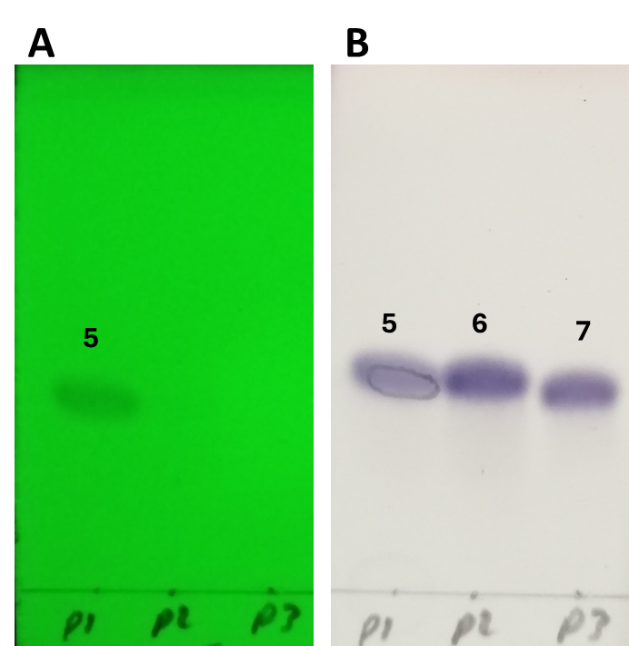


**Figure S32.**  HPLC-RP chromatograms for compounds **5**-**7** after purification detected at 200 nm using the same conditions; **A.** compound **5** eluted at min. 13.739; **B.** compound **6** eluted at min. 13.849; **C.** compound **7** eluted at min. 13.983.


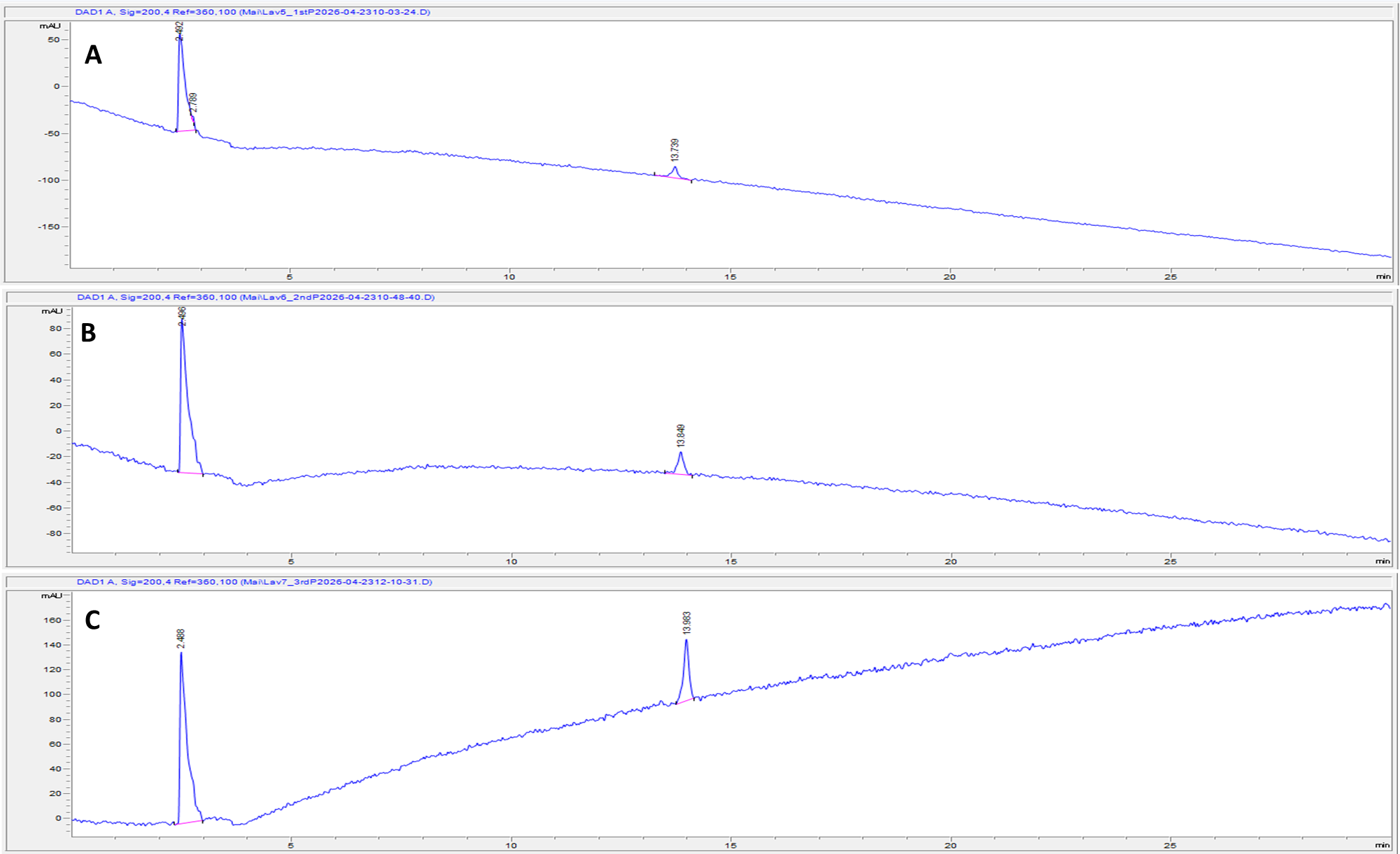


**Figure S33.**  HPLC-RP chromatograms for compounds **5**-**7** after purification detected at 210 nm using the same conditions; **A.** compound **5** eluted at min. 13.739; **B.** compound **6** eluted at min. 13.849; **C.** compound **7** eluted at min. 13.983.


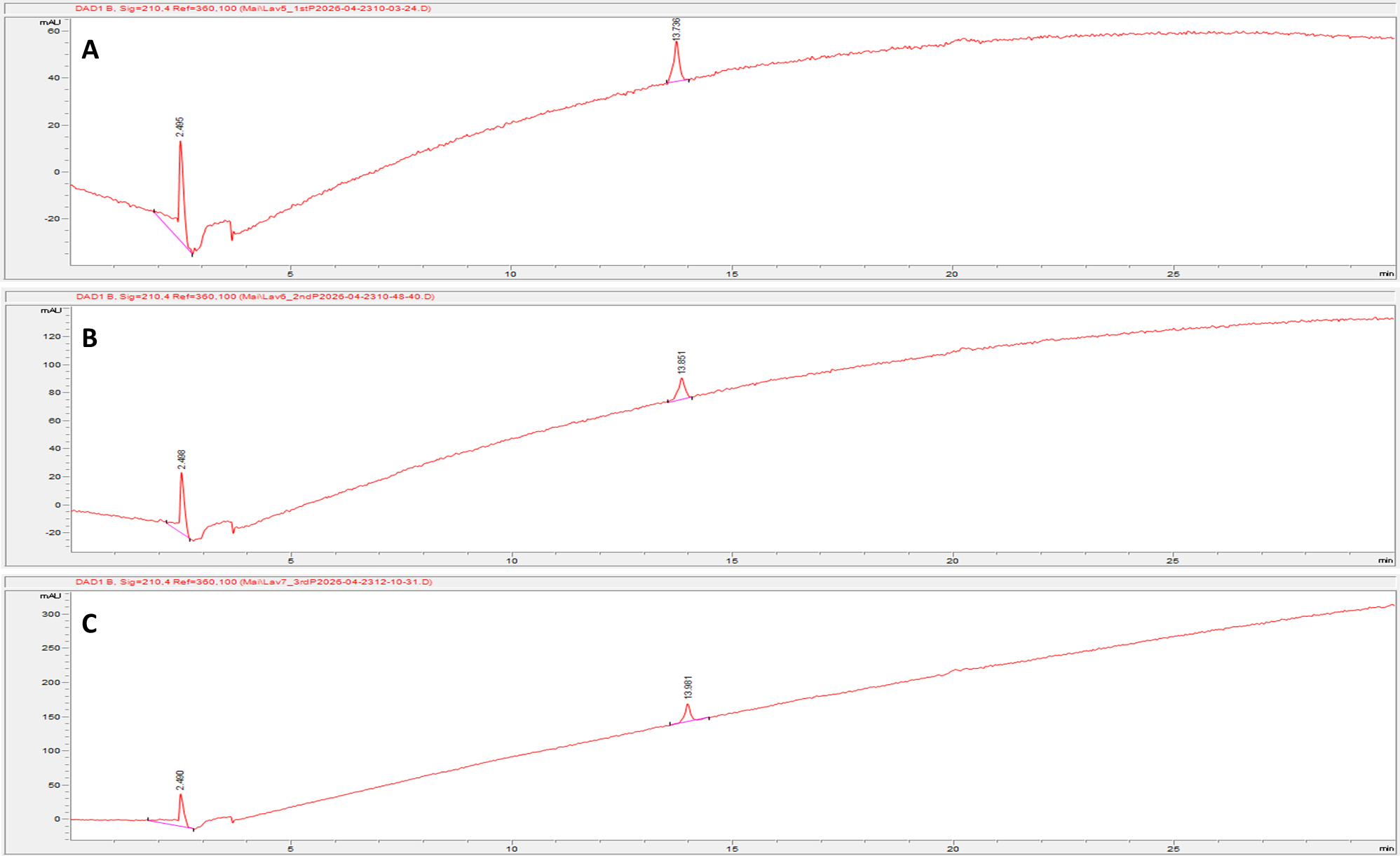

Supplement: Supplementary file 1 — Supplementary Information. [file 41598_2026_51849_MOESM1_ESM.docx]
